# Pipamperone concentrations and organ distribution in postmortem cases with and without overdoses

**DOI:** 10.1007/s00414-026-03830-0

**Published:** 2026-05-09

**Authors:** L. Lucuta, L. Nauroth, J. Hose, M. Juebner, H. Andresen-Streichert

**Affiliations:** Department of Toxicology, Institute of Legal Medicine, Faculty of Medicine and University Hospital, University of Cologne, Melatenguertel 60/62, Cologne, 50823 Germany

## Abstract

Pipamperone is a widely used antipsychotic, which can also be important in death cases. The interpretation of intoxication cases involving pipamperone remains difficult, due to limited postmortem and pharmacokinetic data. Hence, autopsy cases with pipamperone detection were examined regarding the organ distribution and postmortem redistribution (PMR). A liquid-liquid-chromatography with tandem mass spectrometry (LC-MS/MS) method for the detection of pipamperone in postmortem blood was developed and validated. Heart and femoral blood concentrations were determined in 32 autopsy cases, whereby organ distribution was determined in 12 cases. The method was applied to organ homogenates (brain, liver and kidney tissue), other body fluids (urine, cerebral spinal fluid (CSF)) and gastric content. Pipamperone concentrations of 0.82–19 mg/L were detected in femoral blood, 1.0–36 mg/L in heart blood. Heart and femoral blood ratios (H/F ratio) ranged from 0.5 to 3.9 (mean: 1.5 ± 0.8). Among organs, highest concentrations were determined in liver tissue (5.8–315 mg/kg). Concentrations in brain, CSF and urine/kidney were comparatively lower. The H/F ratios as well as the concentration differences in the organs and alternative body fluids lead to the assumption that pipamperone exhibits an insignificant to moderate PMR. A fatal intoxication, primarily caused by pipamperone was assumed in four cases, where the highest concentrations of pipamperone in blood (heart: 13.4–36 mg/L, femoral: 9.4–19 mg/L) were detected. Assuming a possible PMR for pipamperone, the interpretation of autopsy cases remains difficult. Drug concentrations must be assessed with care and an increase of postmortem concentrations must be considered.

## Introduction

Pipamperone is an antipsychotic drug, which is prescribed in all age groups. It is applied for chronic psychosis and has calmative, antidopaminergic and sedative effects and is also suitable for the treatment of insomnia, especially for geriatric patients. The neuroleptic effect is attributed to a blockade of dopamine receptors, whereby pipamperone has a 15-fold higher affinity to D_4_-receptors in comparison to D_2_-receptors. Moreover, it has a high affinity to serotonergic 5-HT_2_ receptors, where it acts as an antagonist [1].

Doses for pipamperone treatment are based on indication and vary between 20 mg (initial dose for insomnia in geriatric patients) to maximum 360 mg per day (for psychosis, separated to three single doses of 120 mg) for adults. After single oral administration of 40 mg average peak plasma concentrations of 0.044 ± 0.017 mg/L were observed [2]. A single oral dose of 120 mg led to plasma concentrations in a range of 0.12–0.50 mg/L [3], whereby steady-state concentrations in patients receiving daily oral doses of 360 mg resulted in plasma concentrations in a range of 0.29–0.39 mg/L [4]. Nevertheless, availability of pharmacokinetic data is still limited, especially for bioavailability and the volume of distribution.

Cases of intoxications with pipamperone have been described in literature, but mostly after concomitant application of other antipsychotics or antidepressants [5, 6]. Bont et al. reported a case of a 16-year-old girl, who survived the ingestion of an unknown quantity of pipamperone, which led to a concentration of 3.2 mg/L in plasma. The patient developed a torsade-de-pointes tachycardia [7]. In general, cardiac complications are mainly described in case of an overdose with pipamperone and are also listed as side effects and symptoms of intoxications in the expert information leaflet. After overdose, cardiac arrest and torsade-de-pointes can occur [8]. Besides cardiac effects, somnolence, convulsive attacks, respiratory arrest, acidosis, cerebral oedema, anoxia, cerebral ischemia and coma have been described in patients with pipamperone overdoses.

Fatal mono-intoxications with pipamperone are rarely found in literature. Henning et al. [9] published three cases and measured femoral blood concentrations of 15, 18 and 39 mg/L, and heart blood concentrations of 15, 20 and 51 mg/L. Concomitant medications were found in each case, however in therapeutic concentrations. These authors also investigated the organ distribution of pipamperone in those cases [8]. Ormandy et al. [10] described a case of a pipamperone intoxication with fatal outcome, with 0.99 mg/L in heart and 1.1 mg/L in femoral blood. It was assumed, that pipamperone either might have caused a convulsive attack or lead to cardiac arrhythmia and thus, contributed to the death.

Overall, data on pipamperone intoxications with and without fatal outcome are limited. Since data on pharmacokinetics also remain incomplete, the interpretation of forensic cases is challenging. Regarding the analysis of postmortem samples, possible postmortem redistribution (PMR) – as supposed by Henning et al. [9] - increases the complexity of interpretation. Accordingly, the present study aims to further contribute to the scientific data regarding pipamperone and thereby support forensic-toxicological case work.

## Materials and methods

### Chemicals and solutions

Pipamperone dihydrochloride (99%, HPLC-grade powder) was purchased from Sigma-Aldrich^®^ (Darmstadt, Germany) and pipamperone-d10 dihydrochloride (manufacturer Toronto Research Chemicals (TRC)), was obtained via LGC Standards Ltd. (Wesel, Germany). Water and acetonitrile (UHPLC-MS grade, Chemsolute^®^) were purchased from Th. Geyer (Renningen, Germany). Formic acid (99%, ULC-MS grade) was obtained from Biosolve (Valkenswaard, The Netherlands). Methanol (UHPLC-MS grade, LiChrosolv^®^) and di-sodium hydrogen phosphate (Na_2_HPO_4_, p.a.) were obtained from Supelco^®^ (Darmstadt, Germany). β-glucuronidase from *E. coli* was purchased from Roche Diagnostics GmbH (Mannheim, Germany). QuEChERS Dispersive Solid Phase Extraction (dSPE) Kits for fatty samples (article number: 5982 − 5121) from Agilent (Santa Clara, USA) were used.

### Calibrators and quality control samples

Calibrator samples were prepared in 200 µL whole blood from volunteers. The calibration ranged from 0.05 to 5.0 mg/L, using ten calibrators (equidistant distribution). Whole blood quality control samples were prepared at concentrations of 0.25, 1.5 and 4.5 mg/L, aliquoted and stored at -20 °C until use. Furthermore, commercially available serum quality control samples were used (TDMC 1/23 (samples A and B) from Arvecon GmbH (Walldorf, Germany), TDMC A: 0.13 mg/L and TDMC B: 0.32 mg/L). The internal standard (IS) solution was prepared in a concentration of 1.5 mg/L in methanol.

### Selection of authentic cases and classification

Real case samples were obtained from autopsies performed in the Institute of Legal Medicine in Cologne between July 2020 and May 2023, in which toxicological tests had been commissioned by the public prosecutor’s office. The routine analyses had already been completed, and all cases were closed. The selection of cases was based on the detection of pipamperone in the ´General Unknown Screening´ (GUS) using a High performance liquid chromatography with diode array detection (HPLC-DAD, Agilent Technologies (Waldbronn, Germany), 1260 series Infinity Binary LC System), which is used for screening of medications and drugs of abuse in death cases. The analysis was performed after liquid-liquid alkaline and acidic extraction. Routine toxicological analyses were completed in all cases. Those consisted of the GUS for medications and drugs of abuse, as well as screening for volatile substances (alcohols), also including blood and, if urine was available, urine alcohol determination. Moreover, analyzer-based immunological testing was performed in each case by using a Tecan Freedom EVOlyzer (Tecan Schweiz AG and Abbott, Männedorf, Switzerland) device with microplate EIA Kits for amphetamines, benzodiazepines, cocaine, methadone, opiates and cannabinoids (Abbot Rapid Diagnostics GmbH, Cologne, Germany)). Subsequent to positive immunological testing, confirmatory chromatographic methods (gas or liquid chromatography coupled with mass spectrometry) were performed in each case. The further selection of cases that should be included in the analyses was performed stepwise. In total, 32 cases were chosen, details are shown in Table 1. An analysis of pipamperone was performed in heart and femoral blood in duplicate. Afterwards, the cases were divided in groups depending on whether the cause of death was unknown (group 1), the cause of death was determined as natural (e.g. heart attack, organ failure, tumor disease) (group 2), or non-natural (e.g. polytrauma, death by hanging, drowning) (group 3). Cases of unknown death (group 1) were selected for further analyses and controlled for completeness of fluids and organ tissue for analyses. Specimens of interests were gastric content, cerebral spinal fluid (CSF), urine or kidney tissue, liver tissue and brain tissue. In a further step, cases with pipamperone concentrations < 0.5 mg/L in femoral blood or those where pipamperone did not relevantly contribute to death, e.g. due to an intoxication with alternative substances, were excluded. Hence, 12 cases were chosen for the analysis of the exhibits mentioned above to assess organ distribution of pipamperone (see Table 1, cases 1–12).

**Table 1:** Detailed information on the selected cases, including gender, age, BMI, relevant autopsy and toxicological findings, case history, PMI and cause of death. Toxicological analyses and determination of BAC were usually performed in femoral blood (exceptions are indicated in the table)

Case	Gender, Age [years]	BMI	Case history	Relevant autopsy findings	Other relevant toxicological findings in blood [mg/L]	PMI [days]	Pipamperone in femoral blood [mg/L]**	Cause of death
1	m, 59	27.2	- psychiatric disease - found dead in flat with stab wound in abdomen	- abdominal stab wound - congestion of inner organs - lung oedema - brain oedema - white substance residues in gastric content - increase in cardiac muscle mass - narrowed coronary vessels	Diazepam ↓ Nordiazepam ↓ Oxazepam ↓ Temazepam ↓ Salicylic acid, Ibuprofen* BAC: n.d.	2–3	14	Heart reinfarction, but possible influence of concomitant pipamperone intoxication
2	m, 44	28.1	- found lifeless, depression, - known cardiac arrhythmia	- congestion of inner organs - lung oedema - brain oedema	Venlafaxine: 0.18 + O-Desmethylvenlafaxine: 0.17 Mirtazapine: 0.034 Bisoprolol: 0.26 Lidocaine* BAC: n.d.	3	9.4	Possible pipamperone intoxication or possible cardiac arrhythmia due to cardiotoxic effects of pipamperone, mirtazapine and venlafaxine
3	f, 22	22.5	-depression -posttraumatic stress disorder - suicidality	- congestion of inner organs - lung oedema - brain oedema	Mirtazapine: 0.35 ↑ Quetiapine: 0.85 ↑ Citalopram: 0.76 ↑↑ Sertraline: 0.62 ↑ Prothipendyl: 1.6 ↑↑ Ibuprofen, Cetirizine * BAC: n.d.	5	12	Polydrug intoxication
4	f, 55	18.8	- schizophrenia and depression - suicide attempt in the past - found dead in bathroom	- congestion of inner organs - lung oedema - brain oedema - white residues in gastric content - artery sclerosis - superficial head injury	Lorazepam: 0.28 ↑ Trimipramine: 0.39 ↑ Zuclopenthixol: 0.045 Pregabalin: 4.9 BAC: n.d.	4	19	Polydrug intoxication
5	m, 40	19.3	- found dead in psychiatric clinic	- congestion of inner organs - cerebral swelling - congestion of heart and heart surrounding vessels	Olanzapine: 1.3 ↑ Flupentixol: 0.043	1	1.4	Polydrug intoxication
6	m, 41	36.6	- posttraumatic stress disorder - alcohol abstinence since 3 years - found dead in flat	- increase in cardiac muscle mass - congestion of inner organs - haemorrhagic lung oedema - brain oedema - drug residues (white tablets) in gastric content and small intestine	Amphetamine: 0.25 ↑ Venlafaxine: 0.49 ↑ O-Desmethylvenlafaxine: 0.007 Quetiapine: 0.33 Olanzapine: 0.029 THC ↓ BAC: 0.52‰	3–4	1.9	Polydrug intoxication
7	m, 70	26.6	- found dead in flat - known alcohol abuse	- increase in cardiac muscle mass and musculature steatosis - extended right atrium - head laceration - inflammation of respiratory passages - blood congestion of inner organs - lung oedema with bronchial mucus	Quetiapine: 0.11 ↑ Citalopram: 0.08 Ibuprofen* BAC: n.d.	N/A	3.0	Cardiac failure with possible contribution of quetiapine and pipamperone
8	f, 76	25.0	- depression - poly-neuropathy - found deceased at the foot of the bed in a nursing home, with a forward-leaning upper body and the head resting on the bed	- ventricular hypertrophy - scarred transformation of the myocardium in the posterior wall of the left ventricle (consistent with prior infarct) - arteriosclerosis - calcification of the aortic valves - early chronic hepatic congestion - cerebral softening due to hypoperfusion (cerebral infarction) - congestion of internal organs - cerebral oedema - hemorrhagic pulmonary oedema	Tilidine: 0.11 Nortilidine: 0.34 ↑ Naloxone (qualitative) Mirtazapine: 0.12 Bisoprolol, Salicylic acid* BAC: n.d.	9	1.5	Suspected cardiac failure and possible contribution of pipamperone due to cardiotoxic effects
9	m, 61	22.1	- found dead in psychiatric clinic	- increase in cardiac muscle mass - narrowed left coronary vessel (25% remaining lumen) - pelvic artery sclerosis - congestion of inner organs - lung oedema - brain oedema	Paliperidone↓ THC↓ Bisoprolol* BAC: n.d.	2	1.0	Coronary heart failure with possible contribution of pipamperone
10	f, 51	25.6	- suicidal thoughts - depression - in psychiatric clinic for alcohol withdrawal - found lifeless in bed	- congestion of inner organs - softened and brittle liver tissue - chronic congestion of liver	Promethazine: 1.2 ↑↑ Mirtazapine: 0.15 7-Aminoclonazepam: 0.028 BAC: n.d.	3	1.0	Polydrug intoxication
11	m, 18	28.3	- cannabis abuse - psychiatric disease - found dead in bed	- congestion of upper body parts - congestion of inner organs - haemorrhagic lung oedema - brain oedema - foam in nostrils and respiratory passages - filled urinary bladder	Cocaine: 5.9 ↑↑ Benzoylecgonine: 5.6 ↑↑ THC: 0.011 Amphetamine: 0.16 Risperidone: ↓ Paliperidone: ↓ Dipyrone ↓ BAC: n.d.	2	1.0	Polydrug intoxication with mainly cocaine and additional contribution of pipamperone and amphetamine
12	m, 60	27.0	- found dead in shelter for homeless individuals	- increased myocardial mass - rounded and enlarged cardiac apex - skull-brain trauma (laceration and contusion) - blood congestion of the heart - stasis of inner organs - hemorrhagic pulmonary edema	Clozapine: 0.65 + Norclozapine: 0.45 ↑↑ Risperidone ↓ BAC: n.d.	3–4	0.83	Suspected death from acute cardiac decompensation due to ethanol-toxic cardiomyopathy with possible contribution of pipamperone and clozapine due to cardiotoxic effects
13	m, 20–40 (person unknown, estimation)	21.3	- jumped from 5th floor, found dead on a canopy	- polytraumatic injuries	THC: 0.025 Promethazine: 0.19 Chlorprothixene: 0.16 Quetiapine ↓ Nordiazepam ↓ BAC: 2.12‰	3	0.22	Multiple trauma
14	f, 81	24.9	- jumped from 5th floor - depression and bipolar disorder	- polytraumatic injuries	Zopiclone: 0.012 Mirtazapine: 0.14 Dipyrone: 31 BAC: n.d.	3	0.82	Multiple trauma
15	f, 41	19.6	- found in burning car	- drastic burning injuries - loss of epidermis - charred musculature - fencing posture - heat-related bone fractures - carbon black in respiratory passages - lung oedema - brain oedema	Sertraline: 0.51 Dipyrone ↓ BAC: n.d. heart blood: COHb: 8% cyanide: 0.01 mg/L	1	0.17	Drastic burning injuries and heat effects
16	f, 66	17.2	- schizophrenia - complained about breathlessness and cough - found dead in flat	- left ventricular hypertrophy - artery sclerosis - brain oedema	Olanzapine: 0.058 Dipyrone* BAC: n.d.	3	0.25	Unknown
17	m, 85	27.2	- femoral neck fracture after fall - death in foster home	- increase in cardiac muscle mass - artery sclerosis - bronchopneumonia - haemorrhagic lung oedema - subdural haematoma - hepatic cirrhosis - inflammation of urinary passages	Morphine: 0.052 (free) Sertraline: 0.075 Dipyrone* BAC: n.d.	5	0.11	Pneumonia with possible influence of respiratory depression from morphine
18	f, 41	35.8	- paranoid schizophrenia - substance abuse, and anxiety disorder - previous suicide attempt via medication overdose **(hospitalization for 6 days)**	- pneumonia following aspiration of gastric contents - intestinal ischemia with surgical removal of large portions of bowel - sepsis - effusions in body cavities - liver and spleen loosened/ disintegrated	Diazepam ↓ Nordiazepam: 0.082 Oxazepam: 0.016 Lorazepam: ↓ Quetiapine: ↓ Olanzapine: ↓ Venlafaxine: ↓ Zuclopenthixol ↓ Opipramol ↓ Ketamine, Propofol, Amlodipine* BAC: n.d.	5	0.46	Septic-toxic multiorgan failure
19	m, 66	27.0	- stationary in psychiatric clinic before death - found dead in flat	- beginning putrefaction - brain oedema - haemorrhagic lung oedema - filled urinary bladder - white residues in gastric content	Amitriptyline: 1.7 ↑↑ Nortriptyline: 0.311 Lorazepam: 0.03 Dipyrone* BAC: n.d.	6–7	0.05	Intoxication with amitriptyline
20	f, 89	13.9	- previous COVID infection - symptoms of stroke (unconscious-ness, respiratory insufficiency), - palliative treatment with morphine (but mistakenly higher dose given) - found dead in retirement home	- increase in cardiac muscle mass - congestion of inner organs - haemorrhagic lung oedema - brain oedema - white residues in gastric content - artery sclerosis	Morphine (free): 0.15 ↑ Mirtazapine ↓ Lorazepam ↓ Dipyrone, Valsartan* BAC: n.d.	2	0.16	Most likely morphine intoxication
21	m, 25	21.7	- repeated suicide announce-ments - found hanging in a public place	- alcohol-aromatic odour during autopsy - strangulation mark at throat - congestion of inner organs - haemorrhagic lung oedema - brain oedema	Tramadol: 0.88 O-Desmethyltramadol: 0.018 THC: ↓ BAC: 0.68‰ (femoral blood) UAC: 1.11‰	1	0.15	Hanging
22	f, 48	24.9	- under partial hospitalization treatment for depressive disorder, with a history of diuretic abuse (following paralysis and speech - disturbances due to severe potassium deficiency - found unresponsive in the prone position at the residential address	- congestion of inner organs - cerebral oedema - haemorrhagic pulmonary oedema - foam in the respiratory passages	Trimipramine: 0.30 Sertraline: 0.22 Lorazepam: 0.036 Furosemide: 1.0 Zopiclone: ↓ Ibuprofen* BAC: n.d.	2	0.77	Death following the long-term administration of furosemide, psychotropic medications may have contributed to death
23	m, 74	18.4	- condition after stroke and car accident, care-dependent - diabetes - dementia - unstable personality disorder - suffers from cardiac arrhythmias - found unresponsive in residence	- stenosis of the coronary arteries - cardioembolic infarcts - cerebellar infarct (not recent) - atrophic brain tissue with enlarged gyri and sulci - gallstones - pneumonia - haemorrhagic brain oedema	Dipyrone: 54 Acetaminophen, Ibuprofen, Diclofenac* BAC: n.d.	5	0.30	Pneumonia after fall and cerebellar infarct
24	m, 50	24.8	- history of frequent hospitalizations due to alcohol intoxications and cirrhosis of the liver - possible suicidal intent - found in an unusual position in the footwell of a vehicle head-down on the passenger side, with the buttocks elevated and legs drawn up in the air	-alcohol-aromatic odour - abdominal ascites - small-nodular cirrhotic and enlarged liver - enlarged spleen	Mirtazapine: 0.041 BAC: 2.91‰ UAC: 4.22‰	2–3	0.36	Positional asphyxiation, possibly favoured through alcoholisation
25	m, 59	29.4	- paranoid schizophrenia - exhibited severe pain symptoms. - COVID-19 disease and experienced physical decline - found unresponsive, lying supine on the floor in psychiatric clinic	- increase in myocardial mass - arteriosclerosis - evidence on early chronic hepatic stasis and chronic cholecystitis - indicators of pneumonia - blood statis of inner organs - cerebral oedema, - pulmonary oedema	Clozapine: 1.0 + Norclozapine 0.63 ↑↑ Amisulprid: 1.3 Tilidine: 29 Nortilidine: 83 BAC: n.d.	3	0.53	Suspected cardiac decompensation associated with COVID-19 infection with possible contribution of clozapine and pipamperone due to cardiotoxic effects
26	m, 58	22.5	- cerebral palsy and blindness - bipolar disorder (lithium medication) - was found to be disoriented and confused in nursing home - fever and elevated inflammatory markers - died in hospital after reanimation attempts **(hospitalization for 7 days)**	- paralytic ileus - flaccid and hypochromic organs - fragility of lung tissue - mucus accumulation in the respiratory passages - darkened lung tissue areas resembling food aspiration - septic splenomegaly - a doughy softening of the liver tissue	Morphine (free): 0.096 Fluoxetine: 0.12 Norfluoxetine: 0.28 Lamotrigin: 6.1 Dipyrone: 16 Quetiapine ↓ Acetaminophen* BAC: n.d. lithium: 1.36 mmol/L (day 1) lithium: 0.5 mmol/L (day 3)	4	0.10	Septic multiorgan failure
27	m, 86	24.2	- depression - found in a forest after being reported missing from psychiatric clinic	- increase in myocardial mass - left ventricular hypertrophy - arteriosclerosis - stenosis of the coronary arteries - congestion of inner organs - cerebral oedema, - filled urinary bladder - bloody contents within the colon, indicating possible gastrointestinal bleeding. - signs of hypothermia - rib fractures, cranial swelling, and subcutaneous hemorrhages on the arms, legs, and chest (injuries were considered to be not directly life-threatening)	Mirtazapine: 0.13 Duloxetin: 0.11 Lamotrigin: 3.0 Paliperidone ↓ BAC: n.d.	4–6	0.05	Consistent with cardiac decompensation and intestinal bleeding
28	f, 83	14.1	- femoral neck fracture, followed by postoperative delirium - suspected dementia - had ceased oral intake	- myocardial hypertrophy - arteriosclerosis - signs of chronic hepatic congestion - atrophy and reduction of cerebral tissue - shrinking of internal organs - overdistension and oedema of pulmonary tissue - suppurative pneumonia	Morphine (free): 0.065 Fentanyl: 0.001 Risperidone: 0.019 Paliperidone: 0.027 Midazolam ↓ Melperone ↓	3	0.07	Multiorgan failure
29	m, 81	27.4	- Parkinson’s disease - falls - agitation - decreased responsiveness, with transfer from a senior center to a hospital - fluctuating delirium - respiratory failure (subsequently leading to circulatory failure)	- increase in myocardial mass - mild arteriosclerosis - signs of chronic hepatic congestion - renal cysts - congestion of internal organs - cerebral oedema, - pulmonary oedema (also with minimal purulent content in the left bronchi)	Levetiracetam: 2.7 BAC: n.d.	3	0.28	Cardiac decompensation due to chronic pre-existing defects of the heart
30	m, 14	45.6	- Prader-Willi syndrome (intractable hyperphagia) - gastrointestinal problems and fever - initial diagnosis of gastroenteritis - septic shock - elevated lactate levels - acidosis - arterial hypotension - died in the pediatric intensive care unit	- cerebral oedema - pulmonary oedema - hemorrhagic infiltrates within the lung tissue - suspicion of myocarditis (hemorrhage within the myocardium) - septic multiorgan failure - hepatic hemorrhage - loosened spleen - bowel contained green, liquid, and blood-tinged contents	S-Amphetamine: 0.063 Midazolam ↓ Acetaminophen* BAC: n.d.	1	1.9	Septic multiorgan failure after *Staphylococcus hominis infection*
31	f, 73	19.3	- in-patient in psychiatric clinic - found in the bathroom of the ward in a hanging position (using a belt attached to the shower rod)	- minimal arteriosclerosis of the aorta - strangulation mark on throat - bilateral fractures of the superior horns of the larynx - congestion of inner organs - cerebral oedema	Mirtazapine: 0.15 Zopiclone ↓ lidocaine, ezetimib*	4	0.24	Hanging
32	m, 51	26.3	- found hanging in residence	- Alcoholic-aromatic odor - fractures of both cornua of the hyoid bone - bleeding on both clavicles - acute congestion of inner organs -cerebral edema	Quetiapine ↓ Zopiclone ↓ salicylic acid* BAC: 0.91‰	5	0.57	Hanging

↑ above therapeutic range (in serum/plasma of living persons); ↑↑ possible toxic concentration ^13^; ↓ below therapeutic range, relevant effect of substance at time point of death was not assumed; *chromatographic indications / substance not relevant to specific case and therefore not quantified; **mean of double determination, *n.d.* not detected, *COHb* carboxyhemoglobin, *UAC* urine alcohol concentration, *THC* tetrahydrocannabinol, *m* male, *f* female; post-mortem interval (PMI) was estimated based on the case specific data, *BMI* body mass index, *BAC* blood alcohol concentration, *UAC* urine alcohol concentration

### Sampling and preparations of samples

All postmortem specimen were collected during the autopsy and then stored at − 20 °C until use. The sample from the liver was obtained centrally. For examination of the brain, the medulla was preserved – if possible, in its entirety. Blood samples were pre-treated with ultrasound for homogenization. Urine samples (2 mL) were treated with 10 µL β-glucuronidase for 24 h at room temperature. In cases where no urine was available, kidney tissue was homogenized with HPLC-grade water (1 + 2 (w/v)) and treated with β-glucuronidase accordingly. Brain and liver tissue as well as gastric contents were homogenized with HPLC-grade water (1 + 9 (w/v)).

200 µL of the respective sample were spiked with 10 µL IS solution. Subsequently, 25 µL saturated Na_2_HPO_4_ solution were added to achieve slightly alkaline conditions. Protein precipitation was carried out using 1 mL of acetonitrile. The sample was centrifuged (1000 g, 3 min) and the clear supernatant was diluted (1:100 (v/v)) in mobile phase (85% LC/MS-grade water, 15% acetonitrile, 0.1% formic acid) afterwards. For organ homogenates (brain and liver tissue), the supernatant was first purified using QuEChERS with dSPE Kit according to the manufacturer´s instruction. The purified samples were then diluted in mobile phase accordingly. Samples with concentrations above the calibrated range were diluted with UHPLC grade water and the sample preparation was carried out as described above.

### LC-MS/MS analysis

Measurements were performed on a 1290 Infinity II liquid chromatograph (Agilent Technologies, Santa Clara, USA) coupled with a QTRAP^®^ 6500 + quadrupole mass spectrometer (AB Sciex, Darmstadt, Germany), which was operated in positive electrospray ionization (ESI) mode. A packed Poroshell 120 SB-C18 column (2.1 × 75 mm, 2.7 μm particle size) (Agilent Technologies, Santa Clara, USA) and a gradient elution with water containing 0.1% formic acid (eluent A) and acetonitrile containing 0.1% formic acid (eluent B) were used. Gradient start conditions were 85%/15% (A/B, (v/v)), linear increasing in a time interval from 0 to 1.5 min to 10%/90% (A/B, (v/v)) and decreasing back to the starting conditions within 1.5–4.5 min. A subsequent re-equilibrating time of 1.5 min resulted in a total run time of 6 min. The column oven was set to 30 °C, the flow rate was 0.3 mL/min and the injection volume 2 µL.

Analytes were detected by multiple reaction monitoring (MRM) using the mass spectrometry (MS) conditions shown in Table 2. Nitrogen was used as nebulizer gas (40 psi), auxiliary gas (70 psi), curtain gas (40 psi) and collision gas (9 psi), source temperature was 500 °C and ion spray voltage was 5500 V.

**Table 2:** Multiple reaction monitoring method with collision energy, resolution, mass transitions and retention times

Analyte	Precursor ion m/z [M + H]^+^	Product ions m/z	Retention time [min]	DP [V]	EP [V]	CE [V]	CXP [V]
Pipamperone	376.2	165.0 123.1 **98.1** 74.9	1.4	46	10	39 67 33 137	16 8 12 10
Pipamperone-d10	386.2	291.1 **165.0**	1.3	46	10	29 39	18 12

* *DP* declustering potential, *EP* entrance potential, *CE* collision energy, *CXP* collision cell exit potential; target ions are depicted in bold

### Creatinine measurement

Quantitative creatinine measurements were conducted on an AU 480 Analyzer (Beckmann Coulter, Krefeld, Germany) by a DRI^®^ Creatinine-Detect^®^ test. The method is based on the Jaffe-reaction.

### Method validation

The method was validated according to the guidelines of the Society of Toxicological and Forensic Chemistry (GTFCh) [4]. Statistical data were evaluated using the Excel-based Valistat^®^ software version 2.04 from Arvecon GmbH (Walldorf, Germany). In brief, validation covered linearity, limit of detection (LOD), limit of quantification (LOQ), accuracy with respect to precision and trueness, as well as recovery and matrix effects.

The selectivity was tested in four postmortem as well as two ante-mortem blood samples. Moreover, selectivity regarding different analytes was tested. Therefore, commercially available quality control samples were used (MassCheck^®^ Neuroleptics 1/Extended (lyophilized serum sample, ChromSystems^®^, Gräfelfing, Germany) and STUP 05/22-A WH (lyophilized whole blood sample, ACQ Science, Rottenburg am Neckar, Germany), containing the most important drugs of abuse, as well as antipsychotics and respective metabolites. For accuracy, whole blood controls (QC_low−high_) and commercially available serum controls (TDMC 1/23 (samples A and B)) were used. LOQ was determined according to the guidelines of the GTFCh by sixfold measurement of the lowest calibrator (alternative method 2). LOD was determined based on signal-to-noise (SN) ratio [11].

### Data evaluation of whole blood and alternative matrices

Quantification of pipamperone in femoral and heart blood was performed using the established whole blood calibration curves. Whole blood and serum quality control samples were analyzed in each run (according to GTFCh guidelines [11, 12]).

Other specimen like urine or CSF were also evaluated using the whole blood calibration curve. Control samples for urine and CSF were prepared in a respective blank matrix, which was obtained from a volunteer (urine) and a resolved death case without pipamperone detection (CSF).

The application of a whole blood matrix calibration for the quantification of pipamperone in brain and liver tissue homogenates was tested in two of the cases, additionally using a standard addition procedure to compare with (data shown in appendix, Table 5). After evaluation (see results section), whole blood matrix calibration was considered suitable for the quantification of pipamperone in organ homogenates.

The total amount of the substance in the gastric contents was calculated by multiplying the measured concentration (mg/L) by the volume of gastric content, which was determined during autopsy.

### H/F ratio calculation and creatinine correction

Heart and femoral blood ratios were calculated for each of the 32 cases, based on respective mean values from duplicate measurements.

Urine concentrations were corrected based on creatinine. Standardization was achieved by using the following formula (1). Reference creatinine value was set as 100 mg/dL:

1 $$\begin{array}{l}Creatinine\:corrected\:concentration\left[\frac{mg}{L}\right]=\\Substance\:concentration\:\left[\frac{mg}{L}\right]\cdot\:\:\frac{Reference\:creatinine\:\left[\frac{mg}{dL}\right]}{Sample\:creatinine\:\left[\frac{mg}{dL}\right]}\end{array}$$

Creatinine correction was not possible in three cases (cases 4, 6 and 7), where kidney tissue was examined instead of urine and in one case (case 9), where sample material was limited.

## Results

### Validation

The validation was successfully performed according to the GTFCh guidelines [11]. The corresponding data for accuracy, matrix effects and extraction efficiency are shown in Table 3. The selectivity of the method was confirmed, as the whole blood samples spiked with antipsychotics and drugs of abuse showed no interference. Linearity of calibration was confirmed by correlation coefficient calculation (R2 > 0.99) and Mandel’s F test. Absence of outliers (Grubbs test) was demonstrated for the calibration series. Homogeneity of variance was not confirmed by the F-test, indicating heteroscedasticity. Therefore, statistical weighting was required (1/x). The LOD and LOQ were determined at 0.01 mg/L and 0.05 mg/L, respectively.

**Table 3:** Validation data for pipamperone – quality control samples

	Trueness (Bias) [%]	Precision [%]	RSD_T_ [%]	RSD_*r*_ [%]	Extraction efficiency [%]	Matrix effects [%]
QC_low_ [0.25 mg/L]	-5.6	6.4	6.5	5.3	88.0 ± 7.8	97.6 ± 3.0
QC_mid_ [1.5 mg/L]	-0.4	5.7	5.8	3.3	91.6 ± 6.1	97.8 ± 2.6
QC_high_ [4.5 mg/L]	4.8	5.9	6.0	3.5	n.t.	n.t.
TDMC A [0.13 mg/L]	0.6	5.9	6.0	4.7	n.t.	n.t.
TDMC B [0.32 mg/L]	4.9	6.2	6.2	5.6	n.t.	n.t.

* *RSD*_T_ time different intermediate precision, *RSD*_r_ repeatability, *QC*_low_ quality control sample (low concentration), *QC*_mid_ quality control sample (medium concentration), *QC*_high_ quality control sample (high concentration), *n.t.* not tested

Standard addition was tested exemplarily for the quantification of pipamperone in brain and liver organ homogenates of two cases. Bias between − 2.8% and 2.6% were observed in comparison to quantification of pipamperone in the tissues via whole blood matrix calibration. Hence, whole blood matrix calibration was used for further data evaluation to minimize preparation times and simplify the quantification method. Corresponding data can be found in appendix (Table 5).

### Heart and femoral blood concentrations

Pipamperone concentrations of 0.82–19 mg/L were detected in femoral blood, 1.0–36 mg/L in heart blood. Heart and femoral blood ratios (H/F ratio) ranged from 0.5 to 3.9 (mean: 1.5 ± 0.8, median 1.32; *n* = 32).

Raw data of measured heart and femoral blood concentrations and corresponding H/F ratios can be found in the appendix (Table 6). One case was excluded from calculation (case 15), since the ratio (H/F = 12) was assessed as an outlier via Grubbs test.

### Organ distribution

The measured concentrations in all matrices of the 12 selected cases are shown in Table 4. The cases were grouped based on femoral blood concentrations of pipamperone for better comparability of the results. The concentration ranges for the groups were > 5 mg/L (group 1), 1–5 mg/L (group 2) and 0.5 – < 1 mg/L (group 3). For gastric content, measured concentrations, volume of gastric content (total volume, V_tot_) and respective total amount of pipamperone are given. Urine concentrations were corrected with respect to creatinine (compare Sect.  2.8).

**Table 4:** Concentrations of pipamperone in postmortem blood samples (heart and femoral blood), urine, cerebral spinal fluid, gastric content and organ homogenates (brain, liver, kidney tissue) in 12 cases

	Group 1				Group 2				Group 3			
	Case 1	Case 2	Case 3	Case 4	Case 5	Case 6	Case 7	Case 8	Case 9	Case 10	Case 11	Case 12
Femoral blood [mg/L]	14	9.4	12	19	1.4	1.9	3.0	1.5	1.0	1.0	1.0	0.82
Heart blood [mg/L]	31	36	13	27	1.5	7.6	1.9	3.0	2.3	1.0	1.7	1.3
Urine [mg/L]	28^#^	6.5^#^	221^#^	n.a.	18^#^	n.a.	n.a.	34^#^	0.84**	0.51^#^	16^#^	8.7^#^
Kidney [mg/kg]	n.a.	n.a.	n.a.	93	n.a.	9.6	9.4	n.a.	n.a.	n.a.	n.a.	n.a.
Liver tissue [mg/kg]	315	283	97	275	30	23	32	23	15.9	5.8	12	18
Brain tissue [mg/kg]	49	28	32	63	1.8	4.7	8.0	2.7	1.4	1.2	1.4	1.1
Cerebral spinal fluid [mg/L]	10	7.8	9.1	14	0.42	1.4	1.2	1.0	0.47	0.33	0.55	0.40
Gastric content [mg/L] Total volume [mL] Total amount [mg]	585 V_tot_ = 650 380	346 V_tot_ = 40 13.8	1062 V_tot_ = 100 106	1492 V_tot_ = 250 373	4.3 V_tot_ = 100 0.43	70 V_tot_ = 350 24.5	16 V_tot_ = 2 0.03	11 V_tot_ = 10 0.11	2.9 V_tot_ = 550 1.5	5.2 V_tot_ = 4 0.02	9.9 V_tot_ = 25 0.24	2.0 V_tot_ = 150 0.3

^#^ creatinine corrected value, **creatinine measurement and thus correction not possible due to limited sample material, *n.a*. not available, *V*_tot_ total volume

## Discussion

### Validation

A method for the quantitative determination of pipamperone in postmortem blood was developed. A less intense target ion (m/z 98.1) was used for quantification, since a better linearity could be achieved over the measuring range.

According to the guidelines of the GTFCh, the signal-to-noise ratio (S/N) at the LOD (limit of detection) should be at least 3:1. The determined signal-to-noise-ratios for both, the target and qualifier ions, were comparatively higher, suggesting that an even lower concentration could likely have been determined as the LOD.

The investigation of linearity demonstrated a proportional relationship between the analytical signal and the concentration. However, reliable results for the lower calibration points could only be obtained by applying a weighting factor of 1/x.

The observed matrix effects of approximately 97% at the two measured QC sample concentrations (0.25 mg/L and 1.5 mg/L) indicate only minimal ion suppression. Considering the standard deviation, the measured matrix effects may also be attributed to measurement uncertainty rather than co-eluting substances. The investigation of the recovery revealed only minor losses during sample preparation. This may, for instance, be due to analyte entrapment during protein precipitation. Based on the consistently good accuracy of all QC samples and the similar matrix effects and extraction efficiencies observed at the above-mentioned concentrations (compare respective data in Table 3), a further determination of matrix effects or extraction efficiency for the other QC samples was not considered necessary.

The method was also suitable for the determination of pipamperone in alternative matrices like urine, CSF and organ homogenates. Control samples for CSF and urine were analyzed using the whole blood calibration curve to assess the suitability of the method for those alternative matrices. A limitation of that procedure is that individual samples for CSF and urine were used as blank matrices for control samples. Since the method was specifically developed and validated for quantitative determination of pipamperone in whole blood, this approach was considered acceptable. However, for routine analyses in urine or CSF, additional measurements using matrices derived from multiple donors would be necessary. Usually, also a calibration with the same matrix as the sample matrix would be preferable, but has to be reasonable with respect to sample preparation time.

For organ homogenates, the use of serum/blood matrix calibrations for the quantification of drugs has been described before and is a commonly used method [13–15]. Experiments concerning the use of a standard addition for the quantification of pipamperone in organ homogenates revealed minor variations (bias − 2.8% to 2.6%) in comparison to the use of a whole blood calibration as reference for quantification in two cases. Accordingly, a whole blood matrix calibration was considered efficient for the quantification of pipamperone in organ homogenates as well.

### Pipamperone blood concentrations and H/F ratios

Pipamperone concentrations in femoral and in heart blood were comparable in some of the cases, showing H/F ratios of 1.0 (6 / 31 cases). Heart and femoral blood ratios (H/F ratio) of > 1.0 were observed in 19 out of 31 cases, expressing higher heart than femoral blood concentration. Femoral blood concentrations were higher in 6 out of 31 cases.

The results of heart and femoral blood measurements indicate that pipamperone may exhibit PMR, since drugs that have a higher H/F ratio (> 1.0) are believed to have greater tendency for PMR [16]. In the present study, H/F ratios between 0.5 and 3.9 (mean: 1.5 ± 0.8, median: 1.3) were observed, excluding one case (case 15, outlier) with a very high ratio of 12. In the latter case, circumstances of death (found in a burning car, severe burning injury) might have contributed to the unusual ratio in comparison to the other ratios. Changes in drug pharmacokinetics of burn patients have been described in literature [17]. Thus, postmortem drug concentrations might also be affected and concentration changes might be unpredictable.

Concerning the H/F ratios of pipamperone, literature data are limited. Henning et al. [9] investigated H/F ratios of 0.8 and 1.3 in cases of assumed fatal pipamperone intoxication and also investigated organ distribution. Based on their data they suggested, that pipamperone may exhibit PMR, although the number of cases in the study was limited (*n* = 3). Brockbals et al. [15] observed an average H/F ratio of 0.9 in two cases (single values of each case not shown in publication).

However, a sole consideration of the H/F ratio is not suitable for reliably assuming PMR, since there are important factors influencing the ratio. These are e.g. the type of drug, its volume of distribution (V_D_), blood-plasma-ratio, dose, PMI and the pKa-value [18]. An incomplete absorption and distribution of the drug prior to death may be of importance as well, since concentrations of a substance are known to be higher in the blood of the arteries than in the blood of the veins, especially short after administration [19, 20]. A diffusion from high drug amounts in gastric content might contribute to higher drug concentrations in heart blood. This has been shown especially for ethanol, but was assumed to be less substantial for drugs [21].

Additionally, it is stated in literature that higher drug concentrations in femoral blood compared to heart blood may be a result of resuscitation [22, 23]. Ratios of 3.8 and 0.5 were observed in two of the examined cases (cases 2 and 30) in the presented study, where resuscitation was documented. The opposing concentration ratios do not fully align with literature, although a higher number of cases where resuscitation occurred should be examined to strengthen this observation.

According to literature, basic drugs with a large volume of distribution (V_D_ > 3 L/kg), which are present in extracellular fluid and sequestered in tissue, have a tendency for PMR [18, 24]. Those substances can show H/F ratios of up to 20. Thus, e.g. antidepressants like amitriptyline exhibit relevant PMR [18]. Conversely, for substances such as acetaminophen (V_D_ = 0.8–1.0 L/kg) and mirtazapine (V_D_ = 10–14 L/kg) [24] a relationship between V_D_ and PMR has not necessarily been confirmed [19]. Only few studies address the pharmacokinetic properties of pipamperone, and the V_D_ respectively. Kloosterbor et al. [25] calculated a mean V_D_ of 451–456 L after a 120 mg dose in healthy volunteers (mean weight 76.8 kg), assuming a bioavailability of 100%. The reported high volume of distribution (≈ 5.9 L/kg) indicates an extensive tissue distribution, which might be another indicator for PMR.

Another factor that has to be taken into consideration is the distribution of a substance between cellular components, particularly red blood cells, and serum/plasma [18]. The blood-plasma ratio of pipamperone was estimated at 0.21 ± 0.03 by Tron et al. [26] by comparing plasma concentrations and dried blood spot concentrations of pipamperone. Postmortem changes such as autolysis and putrefaction lead to a loss of cellular integrity, a decrease in pH, and dissolution of organ structures. As a consequence, concentration gradients between tissues and blood are disrupted [27, 28]. For pipamperone, which is considered to have a low blood-to-plasma concentration ratio, no relevant sequestration in blood cells can be assumed. Hence, the loss of membrane integrity may not result in redistribution from blood cells but rather in the release of the drug from tissues into the vascular compartment.

Overall, the observed variability in H/F ratios may be explained by the rather high V_D_ of pipamperone, together with its low blood-plasma ratio, which facilitates PMR from tissues into heart blood. Regarding higher femoral blood concentrations in comparison to heart blood, local tissue stores around the femoral vein, such as subcutaneous fat and muscle, may release pipamperone postmortem into femoral blood, slightly elevating its concentration. However, redistribution into heart blood from central organs generally exerts a stronger effect.

### Organ distribution

The analysis of 12 selected cases allowed the observation of relationships in the organ distribution of pipamperone.

#### Brain, blood and CSF

In general, little is known regarding the correlation between concentrations in femoral blood and CSF in postmortem toxicology [19]. Hiemke et al. [29] described a rapid distribution of antipsychotics from the blood into the central nervous system, typically resulting in higher concentrations in the brain than in blood. This finding is consistent with the results of the present study. Pipamperone concentrations in brain tissue were higher than in femoral blood and CSF for all observed cases. Henning et al. [9] measured brain tissue concentrations from 41 to 71 mg/kg, which is comparable to the brain tissue concentrations of cases 1–4 in this study, where also comparable femoral blood concentrations were measured (range 28–63 mg/kg). Further information regarding the origin of examined brain tissues in the study by Henning et al. [9] is not available. A significant factor of uncertainty may be variations in concentration across different regions of postmortem organs [30]. Since pipamperone primarily binds to D_4_-receptors, different drug concentrations may be expected in areas with high D_4_-receptor density (e.g., hippocampus, frontal lobe, amygdala) compared to the medulla, which was sampled in this study.

#### Kidney/Urine

Pipamperone is metabolized hepatically and is primarily excreted via the kidneys. Biotransformation via N-dealkylation, keto reduction, piperidine ring hydroxylation, N-oxidation and amide hydrolysis leads to a series of pharmacologically inactive metabolites [3, 4]. For extensively metabolized drugs, the parent compound is usually not detectable in urine or only present at very low concentrations [18, 30]. Koeppel et al. [4] detected unchanged pipamperone in urine. However, data regarding the concentration ratios between the parent drug and its metabolites are not available.

The concentrations of pipamperone in urine and kidney tissue observed in this study showed a discrepancy compared to the values reported by Henning et al. [9], which ranged from 445 to 548 mg/L in urine. The highest measured concentrations in the examined cases were 257 mg/L in urine (Case 3) and 93 mg/kg in kidney tissue (Case 4), whereas the concentrations in the other cases ranged from minimum 0.15 to maximum 31 mg/L. The detected concentrations in urine or kidney homogenate were even below 10 mg/L in more than 50% of the investigated cases. These differences between the measured urine concentrations and those reported in the literature may be attributed to the efficiency of the extraction process or matrix effects. Henning et al. [9] used trimipramine-D3 as an internal standard (IS) and performed standard addition, whereas in this study, pipamperone-D10 was used as IS, and quantification was based on a blood calibration. Another factor might be a missing creatinine correction of Henning et al. in comparison to the herein presented data.

The comparability of the data might also be restricted by the smaller number of cases investigated by Henning et al. [9]. However, urine concentrations can be affected by various factors and remain a qualitative parameter for interpretation, which cannot be compared directly among cases.

#### Gastric content

Data for pipamperone concentrations in gastric content are not available in literature for comparison. In general, the highest drug amounts were found in gastric contents of group 1, especially in cases 1 and 4 (380 mg and 373 mg in total). The presence of substantial amounts of pipamperone in the gastric contents in those cases indicates that a portion of the administered dose remained unabsorbed at the time of death. Corresponding femoral blood concentrations (14 and 19 mg/L) reflect the fraction of the substance that has already been absorbed and systemically distributed. Heart blood concentrations (27 and 31 mg/L) are higher than femoral concentrations in both cases, which would be consistent with PMR.

#### Liver

The highest measured concentrations in liver tissue in the four cases of group 1 (97 to 315 mg/kg) were exceeding those reported in literature in three of the cases. Hence, Henning et al. [9] detected concentrations ranging from 91 to 190 mg/kg.

With respect to the concurrently rather high pipamperone amounts in the gastric content in three of the herein presented cases (106–380 mg), a diffusion from gastric content to liver might have occurred. Diffusion from gastric content to liver has been described in literature for other drugs [19, 21]. To the best of the authors’ knowledge, there is no evidence that pipamperone undergoes enterohepatic circulation that could account for increased liver concentrations.

Variations in sampling site (sampling site not published by Henning et al. [9]) might also account for these differences. The liver is interspersed with connective tissue and enclosed by the liver capsule. This can result in different proportions of functional hepatocytes depending on location of tissue sampling and possible drug concentration differences depending on the sampling site. A study on diffusion from gastric content with amitriptyline, acetaminophen and lithium in a human cadaver model revealed high, site-dependent, drug concentrations in the lungs and liver. Hence, e.g. amitriptyline concentrations, which has most comparable physicochemical properties to pipamperone among the investigated substance, were 9-fold higher in the left anterior lobe in comparison to the right lobe [21].

#### PMI

The influence of the PMI and sampling time point after death on postmortem concentrations in blood and tissue has been described in literature, especially for antidepressants and antipsychotics [15, 31, 32], whereby concentration increases and decreases can occur. Brockbals et al. [15] investigated time-dependent concentration changes for pipamperone in femoral blood in two cases. One case did not show concentration changes, while an increase of the pipamperone concentration of 21% was observed in the second case (data on PMI not shown in respective cases). The PMI was estimated between 1 and 9 days among the presented cases of this study. At a short PMI of approximately one day, H/F ratios of 0.5–1.8 (*n* = 3) were observed for pipamperone. At a PMI of 2–3 days, ratios ranged from 0.7 to 3.9 (*n* = 14). At a PMI of over 3 to 4 days, ratios ranged from 0.9 to 3.8 (*n* = 5). This heterogeneity of results was also observed at higher PMIs (over 4 days) among the presented cases. Thus, the PMI might contribute to the H/F ratio, but influences on the H/F ratio are rather be multifactorial.

## Conclusion

A method for the quantitative determination of pipamperone in postmortem blood has been developed and validated. The method was also applicable to organ homogenates and urine samples. Overall, the measurements of pipamperone in postmortem heart and femoral blood in 32 cases complement available data on pipamperone concentrations in postmortem cases. The calculated H/F ratios of 31 cases and the measured organ concentrations in 12 cases additionally support the assumption that pipamperone exhibits an insignificant to moderate PMR. These extensive data will help forensic toxicologists to evaluate analytical results more reliably in the future.

## Data Availability

The datasets generated during and/or analyzed during the current study are available from the corresponding author on reasonable request.

## Appendix

**Table 5:** Comparison of measurements of two organ specimen (brain and liver) from two cases using standard addition and whole blood matrix calibration by calculation of bias

Case	Specimen	Standard addition[mg/kg]	Whole blood calibration[mg/kg]	Bias [%]
1	Brain	47.8	47.2	-0.6
	Liver	388	366	-2.8
5	Brain	1.86	1.90	1.0
	Liver	26.8	28.2	2.6

**Table 6:** Meanpipamperone concentrations in heart and femoral blood (measurement in duplicate) and corresponding H/F ratio

Case	Heart blood[mg/L]	Femoral blood[mg/L]	H/F ratio	
1	31	14	2.2	2-3
2	36	9.4	3.8	3
3	13	12	1.0	5
4	27	19	1.3	4
5	1.5	1.4	1.0	1
6	7.6	1.9	3.9	3-4
7	1.9	3.0	0.6	n/A
8	3.0	1.5	1.9	9
9	2.3	1.0	2.3	2
10	1.0	1.0	1.0	3
11	1.7	1.0	1.6	2
12	1.3	0.83	1.5	3-4
13	0.37	0.22	1.6	3
14	1.7	0.82	2.1	3
15	2.0	0.17	12*	1
16	0.27	0.25	1.0	3
17	0.13	0.11	1.1	5
18	0.42	0.46	0.9	5
19	0.05	0.05	0.9	6-7
20	0.15	0.16	0.9	2
21	0.27	0.15	1.8	1
22	2.2	0.77	2.8	2
23	0.34	0.30	1.1	5
24	0.57	0.36	1.5	2-3
25	0.41	0.53	0.7	3
26	0.18	0.10	1.7	4
27	0.05	0.05	1.0	4-6
28	0.09	0.07	1.2	3
29	0.32	0.28	1.0	3
30	1.0	1.9	0.5	1
31	0.55	0.24	2.2	4
32	0.76	0.57	1.3	5
Mean ± SD			1.5 ± 0.8	
Median			1.3	

*Outlier. Excluded from calculation of mean, median and standard deviation (SD), *H/F ratio* Heart/Femoral blood ratio, *PMI *= post mortem interval, *N/a *= information not available
